# Class 3 Semaphorin Mediates Dendrite Growth in Adult Newborn Neurons through Cdk5/FAK Pathway

**DOI:** 10.1371/journal.pone.0065572

**Published:** 2013-06-10

**Authors:** Teclise Ng, Jae Ryun Ryu, Jae Ho Sohn, Terence Tan, Hongjun Song, Guo-li Ming, Eyleen L. K. Goh

**Affiliations:** 1 Program in Neuroscience and Behavioral Disorder, Duke-NUS Graduate Medical School, Singapore, Singapore; 2 Department of Physiology, Yong Loo Lin School of Medicine, National University of Singapore, Singapore, Singapore; 3 Institute for Cell Engineering, Johns Hopkins University School of Medicine, Baltimore, Maryland, United States of America; 4 Department of Neurology, Johns Hopkins University School of Medicine, Baltimore, Maryland, United States of America; 5 The Solomon H. Snyder Department of Neuroscience, Johns Hopkins University School of Medicine, Baltimore, Maryland, United States of America; Institut de la Vision, France

## Abstract

Class 3 semaphorins are well-known axonal guidance cues during the embryonic development of mammalian nervous system. However, their activity on postnatally differentiated neurons in neurogenic regions of adult brains has not been characterized. We found that silencing of semaphorin receptors neuropilins (NRP) 1 or 2 in neural progenitors at the adult mouse dentate gyrus resulted in newly differentiated neurons with shorter dendrites and simpler branching *in vivo*. Tyrosine phosphorylation (Tyr 397) and serine phosphorylation (Ser 732) of FAK were essential for these effects. Semaphorin 3A and 3F mediate serine phosphorylation of FAK through the activation of Cdk5. Silencing of either Cdk5 or FAK in newborn neurons phenocopied the defects in dendritic development seen upon silencing of NRP1 or NRP2. Furthermore, *in vivo* overexpression of Cdk5 or FAK rescued the dendritic phenotypes seen in NRP1 and NRP2 deficient neurons. These results point to a novel role for class 3 semaphorins in promoting dendritic growth and branching during adult hippocampal neurogenesis through the activation of Cdk5-FAK signaling pathway.

## Introduction

Active neurogenesis in mammals occurs throughout life in the subventricular zone (SVZ) of the lateral ventricle and in the subgranular zone (SGZ) of the dentate gyrus in the hippocampus [1], [2]. Extrinsic factors regulating the development of neural progenitors within the SGZ include Shh [3], [4], Wnt [5], EGF [6], FGF-2 [7] and VEGF [8]. While it is well known that these external factors play important roles in proliferation, maintenance and survival of neural progenitors in the neurogenic niches, there remains a paucity of studies on how extrinsic factors shape intricate dendritic and axonal branching patterns that are critical for functional integration into existing adult neural networks. It is thus an intriguing and untested possibility that classical embryonic guidance cues can be co-opted for targeting and synaptic connections made by neurons born in the adult life.

Semaphorins comprise of a large family of conserved proteins that function as guidance cues in diverse organisms. Classes 1 and 2 semaphorins have been described only in invertebrates. Classes 3 to 7 are found in vertebrates while class 8 encodes viral semaphorins [9]. Class 3 semaphorins are well characterized in the developing nervous system, and were initially identified to be axon repulsive cues. Subsequently, they were demonstrated to have both attractive and repulsive effects in various systems [10]. Class 3 semaphorins can act as repulsive or attractive guidance cues for subpopulations of neurons depending on their spatial and temporal distributions [11], [12]. Sema3A regulates dendritic growth of cortical pyramidal and hippocampal CA1 neurons during development [13], [14], [15]. In addition, Sema3A and 3F play important roles in dendritic spine maturation [16], [17] and synaptic transmission [18], [19]. More recently, it was reported that Sema3A plays a critical role in axonal-dendritic polarity in neurons [20], [21]. Despite their better-known functions during development, the mechanisms of class 3 semaphorins signaling and their effects in the adult central nervous system remained largely unknown. However, the presence of Sema3A and 3F and their receptors (including NRP1 and 2) throughout adulthood suggests possible functional roles of Sema3A and 3F in the adult nervous system [10].

Previous studies on semaphorins were mainly focused on their roles in developing or mature neurons using neuropilin knockout mice. Both NRP1 and NRP2 knockout mice tend to be embryonic lethal or die young [22], [23], [24]. This limitation has hindered the investigation of the function of semaphorins in adult brains. Using well established retrovirus-mediated gene-transduction to specifically label newborn neurons allow us to overcome this limitation and to address whether semaphorin signaling regulates the development of neuroprogenitor cells in the adult animal. Here we show that retroviral-mediated knockdown of NRP1 and -2 severely perturbed the dendritic development of newborn neurons in the subgranular zone of adult mouse hippocampus. Previous studies showed semaphorin induced tyrosine phosphorylation of focal adhesion kinase (FAK) is critical for axon collapse and dendritic growth [15], [25], [26]. We found tyrosine phosphorylation on Tyr 397 is essential for dendritic growth in adult newborn neurons *in vivo*. In addition, we uncovered that semaphorin-induced serine phosphorylation of FAK via Cyclin-dependent kinase (Cdk5) plays a previously unreported role in regulating the dendritic development of newborn neurons in the developing and adult brains.

## Results

### Neuropilin-1 and -2 Regulates the Dendritic Development of Newborn Neurons in Adult Hippocampus

**Figure 1:**
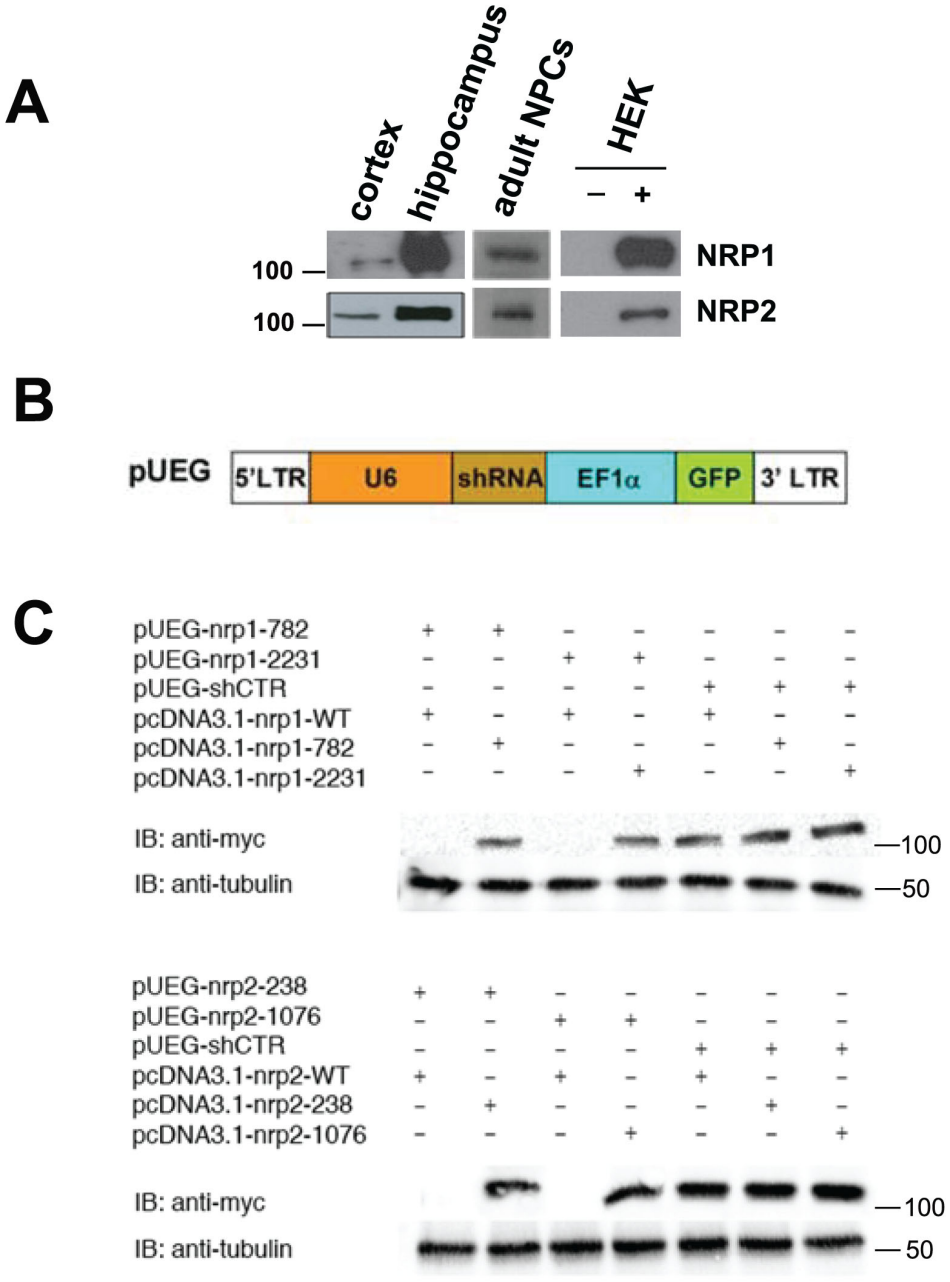
NRP1 and NRP2 are expressed in adult neural progenitor cells and can be knocked down by NRP1 and NRP2 shRNA respectively. (A) Representative western blots showing that NRP1 and NRP2 are expressed in cortical and hippocampal primary neuronal culture and adult hippocampal neural progenitor cells (NPCs). HEK 293 cells were overexpressed with NRP1 (‘+’, upper panel) and NRP2 (‘+’, lower panel) and probed with anti-NRP1 and anti-NRP2 respectively. Untransfected HEK 293 cell lysate (‘−’, upper and lower panels). (B) A schematic diagram of retroviral constructs co-expressing GFP under EF1-α promoter and shRNA driven by the U6 promoter. (C) Retroviral constructs expressing different shRNAs were co-transfected with the indicated myc-tagged overexpression constructs into 293T cells. Western Blot analysis showed effective knockdown of wild-type NRP1 and NRP2 by shRNAs for NRP1 and NRP2. pcDNA3.1-nrp1-782 and pcDNA3.1-nrp1-2231 express NRP1 that harbor silent mutations rendering them are resistant to knockdown by pUEG-nrp1-782 and pUEG-nrp1-2231, respectively. pcDNA3.1-nrp2-238 and pcDNA3.1-nrp2-1076 are resistant to knockdown by pUEG-nrp2-238 and pUEG-nrp2-1076, respectively.

**Figure 2:**
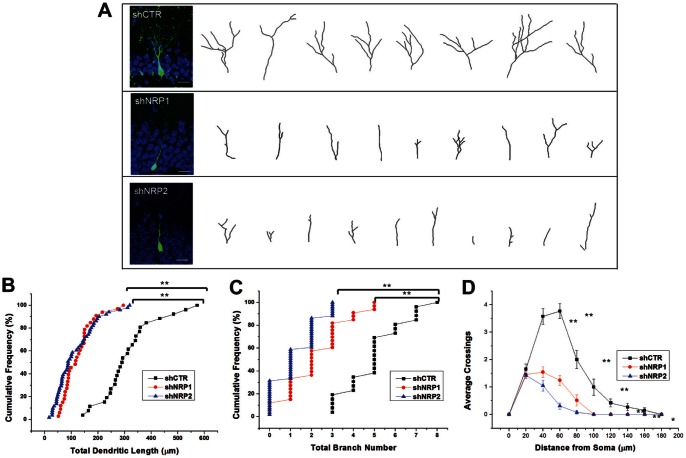
NRP1 and NRP2 regulated dendritic morphology of newborn DGCs in adult brain. (A) Representative images and tracings of dendrites of control, NRP1 or NRP2-shRNA expressing DGCs at 14 dpi (Scale bar, 20 µm). (B, C) Quantification of total dendritic length and branch number of newborn DGCs. Each symbol represents a single DGC. (**P<0.01, 1-way ANOVA with Newman-Keuls’ post hoc test). (D) Graph showing dendritic complexity of GFP^+^ DGCs. Values represent mean ± s.e.m. (**P<0.01, *P<0.05, 1-way ANOVA with Newman-Keuls’ post hoc test).

NRP1 and NRP2 are expressed in cortical and hippocampal primary neuronal culture and adult hippocampal neural progenitor cells (NPCs) (Fig. 1A). To determine the role of Sema3A/NRP1 and Sema3F/NRP2 signaling in adult neurogenesis, we employed a retrovirus-mediated method for birth-dating and genetic manipulation of individual newborn neurons in the adult mouse dentate gyrus. The defective surrounding cells in homozygous knockout mice might affect the development of newborn cells and thus *in vivo* single-cell labeling in adult hippocampus allowed us an unbiased assessment of the new born neurons without confounding developmental defects. Retroviral constructs were engineered to co-express enhanced green fluorescent protein (GFP) and gene specific shRNA to knock down either NRP1 or -2 (Fig. 1B). These shRNAs specifically knocked down the expression of endogenous NRP1 and -2 in primary neurons (Fig. S1) and exogenous NRP1 or -2 expressed in HEK293 cells (Fig. 1C) but were unable to knockdown RNAi refractory forms of NRP1 and -2 (Fig. 1C). We stereotaxically injected high titers of engineered retroviruses into the hilar region of the adult C57BL/6 mouse hippocampus to infect proliferating neural progenitors *in vivo*. Mice were analyzed 14 days post-infection (dpi). Representative images of the dendritic arborization of these labelled neurons are shown in Figure 2A. Interestingly, neurons expressing shNRP1 or shNRP2 exhibited significantly shorter total dendritic length (Fig. 2B) and total branch number (Fig. 2C) compared to neurons expressing shCTR at 14 dpi. Sholl analysis further demonstrated a decrease in the dendritic complexity of these neurons (Fig. 2D). Therefore, both NRPs are essential for the proper dendritic development of adult neurons. In addition, NRP1 or NRP2 knockdown *in vivo* do not appear to affect neuronal fate specification during adult hippocampal neurogenesis. All retroviral-infected neurons are positive for DCX and Prox1, which are markers for immature neurons and dentate granule cells respectively (Fig. S2).

**Figure 3:**
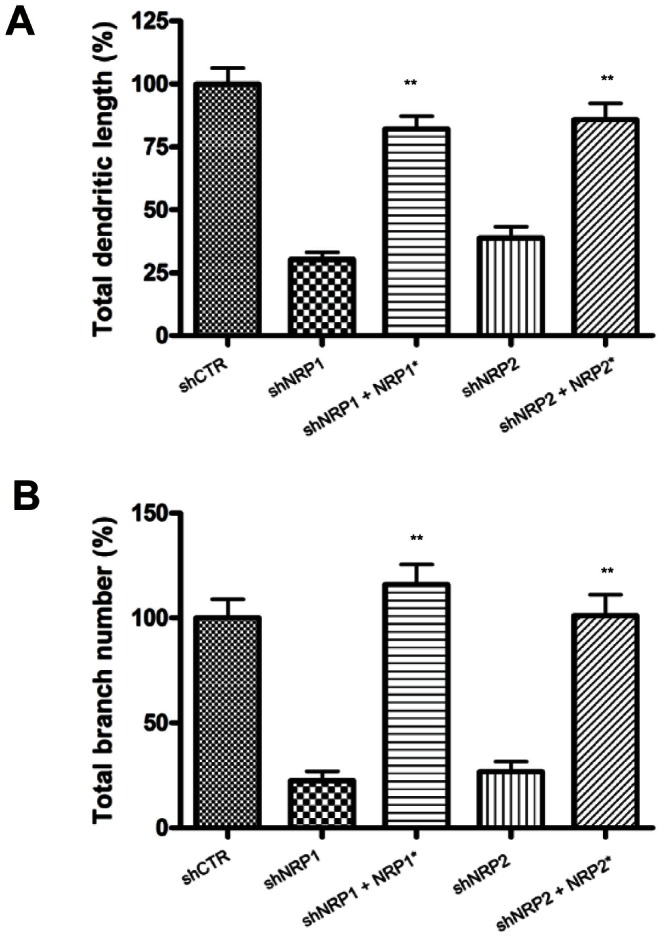
Overexpression of shRNA-resistant NRP1 and NRP2 rescues phenotype induced by NRP1 and NRP2 shRNA. Mixture of retrovirus expressing the indicated constructs was injected into dentate gyrus. Quantification of total dendritic length (A) and branch number (B) of newborn DGCs at 14 DPI. 30–50 neurons from each group were used for analysis. (**P<0.01, 1-way ANOVA with Newman-Keuls’ post-hoc test). NRP1*: NRP1 shRNA-resistant NRP1, NRP2*: NRP2 shRNA-resistant NRP2.

To confirm the specificity of shRNA manipulations *in vivo*, we investigated if the dendritic phenotypes of NRP1 and -2 knockdown neurons could be reversed or rescued by cDNA encoding for shRNA-resistant forms of NRP1 and -2 respectively. These mutated NRP1 and NRP2 are resistant to knockdown by the shRNAs used (Fig. 1C) and could completely rescue the dendritic phenotypes of the neurons deficient in NRP1 or NRP2 (Fig. 3) respectively. These results demonstrated the specificities of the shRNA used and confirmed the role of NRP1 and 2 in dendritic outgrowth of adult-born neurons *in vivo*.

### Neuropilin-1 Mediated Dendritic Development of Newborn Neurons is Independent on Vascular Endothelial Growth Factors (VEGF)

**Figure 4:**
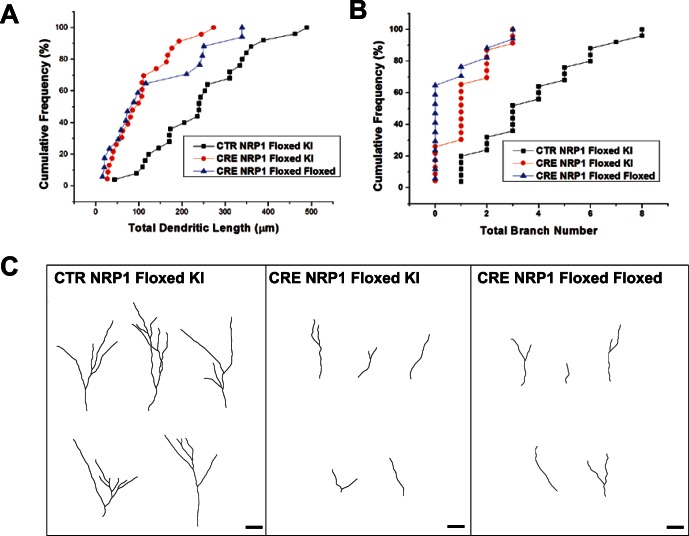
Single cell knockout of NRP1 in neural progenitors results in neurons with impaired dendritic formations is independent of vascular endothelial growth factor (VEGF)-binding of NRP1. (A) Graphs showing quantification of total dendritic length (A) and branch number (B) of newborn DGCs expressing GFP (CTR) or Cre-recombinase (CRE) at 14 DPI in NRP1 conditional knockout mice (NRP1 Floxed/Floxed) or mice expressing one single copy of Floxed-NRP1 and a knockin (KI) of an altered ligand binding site variant of Nrp1 (NRP1 Floxed/KI). (C) Representative tracings of dendritic morphology of adult-born neurons from the above indicated groups. (Scale bar = 20 µm).

Since NRP1 can bind to VEGF [27], we next sought to prove that these phenotypes are not linked to the function of VEGF. We expressed Cre recombinase (Cre) in neural progenitor cells in NRP1 conditional knockout mice. These conditional NRP1 mice (Floxed/Floxed) expressed NRP1 flanked by loxP sites. To ensure the efficiency of Cre and VEGF-binding of NRP1 are not contributing to our observed dendritic phenotypes, we also used another mice expressing one single copy of Floxed-NRP1 and a knockin (KI) of an altered ligand binding site variant of Nrp1 (Floxed/KI) (Fig. 4). This NRP1 variant lost the ability to bind to Sema3A but has intact VEGF binding site [27]. The expression of Cre in these single neuroprogenitor cells results in newborn neurons with defective signaling from only Sema3A but not VEGF. Knockout of NRP1 in individual neural progenitors resulted in impaired dendritic formations, confirming our results using shRNA against NRP1. These observations provided direct evidence that the dendritic phenotypes of NRP1 deficiency we observed was not linked to the function of VEGF and both NRPs are essential for the proper dendritic development of adult neurons.

### Semaphorin 3A or 3F Stimulates Dendritic Growth in Cultured Hippocampal Neurons

**Figure 5:**
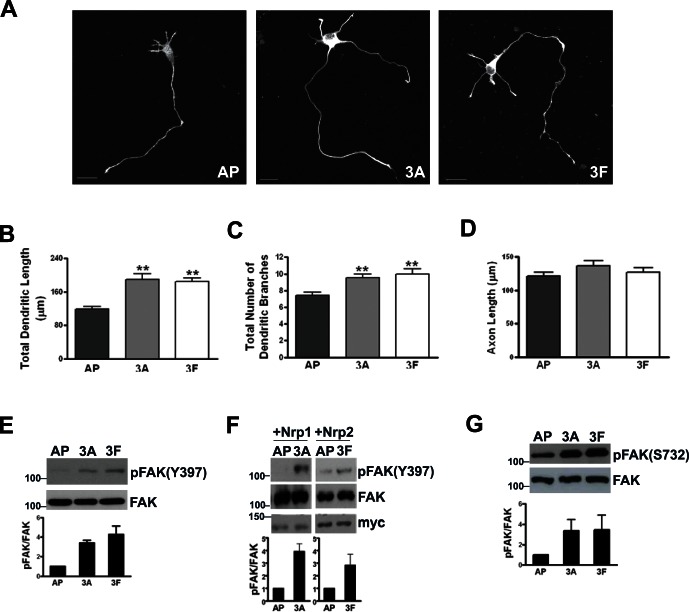
Semaphorin 3A/3F increased dendritic growth in primary neurons and induce tyrosine and serine phosphorylation of FAK. (A) Representative images showing neurons treated with AP-control (AP), Sema 3A-AP (3A) and 3F-AP (3F). Scale bar = 20 µm. (B, C and D) Graphs showing total dendritic length (B), total number of dendritic branches (C) and axon length (D) of primary hippocampal neurons treated with AP control, Sema3A or Sema 3F. (E, F and G) Representative blots showing phosphorylation of FAK in primary neurons (E and G) and 293T cells (F) treated with AP control, Sema3A or 3F. Graph displays change relative to total FAK and values are mean ± s.e.m, n = 3 independent experiments. 40–50 neurons from each group were used for analysis. Values represent mean ± s.e.m. (**P<0.01, 1-way ANOVA with Newman-Keuls’ post-hoc test).

Since primary neurons and adult-born neurons shared many signaling mechanisms, we explored the signaling mechanisms underlying semaphorin regulation of dendritic growth using primary hippocampal neurons that allow isolation of bigger amount of cells as a model system. We then investigated if the same signaling mechanism is conserved in adult-born neurons *in vivo*. In primary hippocampal cultures, alkaline phosphatase (AP) fused semaphorin 3A (AP-Sema3A) treatment at 2 DIV (days in vitro) for 16 hrs increased the total dendrite length by 60% at 189.10±13.59 µm, as compared to 118.30±6.55 µm in AP-treated control neurons (Fig. 5A, B). Similarly, AP-fused semaphorin 3F (Sema3F) treatment also increased the total dendrite length (184.30±8.64 µm). The number of branches per neuron was also significantly increased upon Sema3A or Sema3F treatment (Fig. 5C). Axonal length, on the other hand, was not significantly affected by treatment with either Sema3A or 3F (Fig. 5D).

### The Phosphorylation of FAK Regulates Dendritic Outgrowth in Cultured Hippocampal Neurons

**Figure 6:**
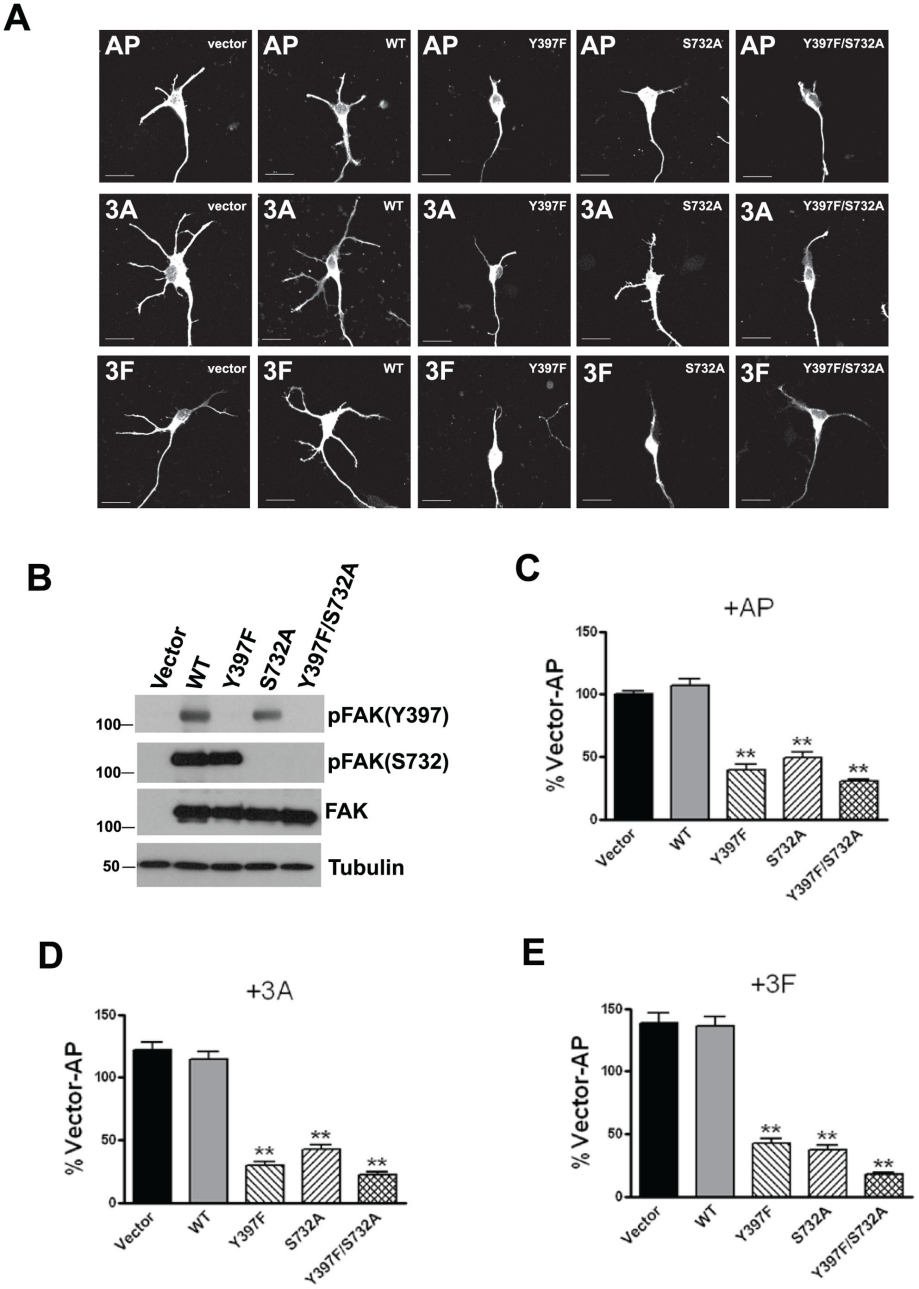
Expression of FAK phosphorylation-defective mutants in primary neurons impaired semaphorin-induced dendritic growth. (A) Representative images of primary hippocampal neurons transfected with vector, wild-type FAK (WT), Y397F FAK (Y397F), S732A FAK (S732A) and Y397F/S732A FAK (Y397F/S732A). Neurons were treated with AP control (AP), 3A-AP (3A) or 3F-AP (3F). Scale bar = 20 µm. (B) HEK293T cells transfected with the indicated constructs encoding FAK or its mutants validating the phosphorylation status of these constructs. (C, D and E) Graphs showing percentage dendritic growth of primary hippocampal neurons expressing FAK mutant constructs as indicated, treated with AP control (C), Sema3A (D) or 3F (E), with percentages normalized to Vector+AP. 30–100 neurons from each group were used for analysis. Values represent mean ± s.e.m. (**P<0.01, 1-way ANOVA with Newman-Keuls’ post-hoc test).

We next examined the potential involvement of FAK signaling in semaphorin-mediated dendritic growth in cultured hippocampal neurons. Tyrosine residue (Tyr) 397 and serine residue (Ser) 732 were both phosphorylated in response to Sema3A or 3F in cultured hippocampal neurons (Fig. 5E, G) and HEK293 cells (Fig. 5F). Since HEK293 cells express endogenous Plexin As and L1 [25], [28], but not NRP1 and -2, we overexpressed NRP1 or NRP2 for Sema3A or Sema3F stimulation respectively. These results indicated that Sema3A and 3F signaling led to tyrosine and serine phosphorylation of FAK in primary hippocampal neurons and that phosphorylation of these two residues might be involved in dendritic growth regulated by Sema 3A and 3F. To dissociate the role of individual critical tyrosine and serine residues in dendrite growth, single tyrosine mutant FAK-Y397F, single serine mutant FAK-S732A and the double mutant, FAK-Y397F/S732A, were generated and overexpressed in HEK293 cells (Fig. 6B). Mutation of S732 did not affect phosphorylation at Y397, and S732 phosphorylation was also not affected in FAK-Y397F expressing cells (Fig. 6B), indicating that phosphorylation of serine and tyrosine residues of FAK were regulated independently. Expression of these phosphorylation deficient FAK mutants decreased basal dendritic growth of hippocampal neurons and also abolished dendritic growth induced by Sema3A or 3F (Fig. 6A, C, D and E). Quantitatively, dendritic growth inhibition by the double mutant FAK-Y397F/S732A was similar to that seen with each single mutant. These results indicated that phosphorylation of both tyrosine and serine residues of FAK were required for dendritic growth induced by Sema3A or 3F. In particular, the involvement of serine phosphorylation of FAK in dendritic growth by Semaphorin led us next to explore upstream signaling molecules of FAK in Semaphorin signaling pathway.

### The Phosphorylation of FAK is Dependent on Cyclin Dependent Kinase 5 (Cdk5)

**Figure 7:**
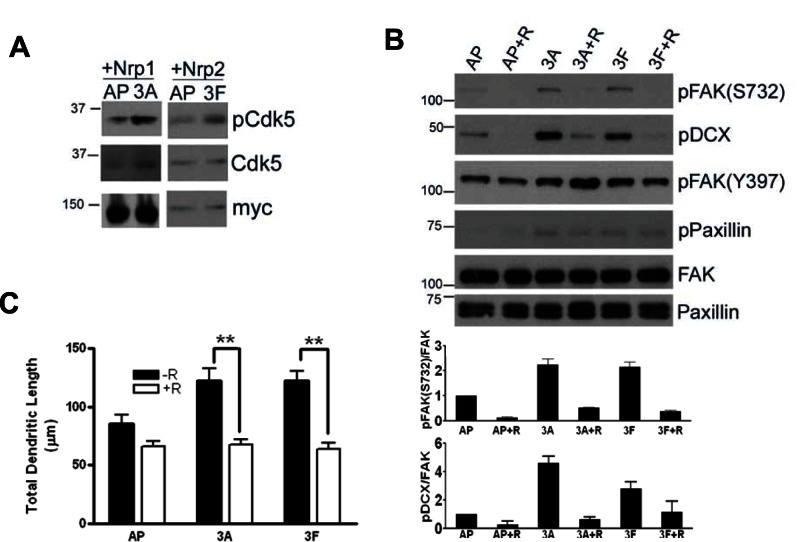
Cdk5 was required for regulating dendritic morphology of primary neurons. (A) Representative blots showing phosphorylation of Cdk5 in HEK293T cells transfected with NRP1 or 2 as indicated, treated with AP control, Sema3A or 3F. (B) Representative blots showing phosphorylation of FAK, paxillin and DCX in primary hippocampal neurons pretreated with vehicle (−R) or roscovitine (+R) prior to AP-control, Sema3A or 3F treatment. Graphs displays changes relative to FAK and values are mean ± s.e.m, n = 3 independent experiments. (C) Graph showing percentage dendritic growth of primary hippocampal neurons treated with vehicle (−R) or roscovitine (+R) together with AP, Sema3A or 3F. 50 neurons from each group were used for analysis. Values represent mean ± s.e.m. (**P<0.01, 2-way ANOVA with Bonferroni’s post-hoc test).

Cdk5 mediates regulation of dendrite orientation by Sema3A in the cerebral cortex [29]. To determine if Cdk5 is involved in Sema3A or 3F-induced dendritic growth, we examined the activation of Cdk5 upon Sema3A or 3F treatment in HEK 293T cells transfected with NRP1 or 2. Addition of Sema3A/3F resulted in significant activation of Cdk5 as compared to control (Fig. 7A). The phosphorylation of DCX, another known substrate of Cdk5, confirmed Cdk5 activation upon treatment with Sema3A or 3F in hippocampal neurons (Fig. 7B). Serine phosphorylation of FAK and DCX induced by Sema3A or 3F were decreased upon treatment with roscovitine, which inhibits the activity of Cdk5 (Fig. 7B). As expected, tyrosine phosphorylation of FAK and paxillin were not affected by roscovitine treatment (Fig. 7B). These results suggested that FAK acted downstream of Cdk5 in Sema3A or 3F signaling. Next, we examined if the activation of Cdk5 was required for the increased in dendritic growth upon Sema3A or 3F treatment. Roscovitine treatment inhibited Sema3A or 3F-mediated increase in total dendritic length of the cultured hippocampal neurons (Fig. 7C) indicating that Cdk5 played a role in dendritic growth regulated by Sema3A or 3F, likely through the activation of FAK.

### Overexpression of FAK Rescues the Inhibition of Dendritic Outgrowth by Roscovitine

**Figure 8:**
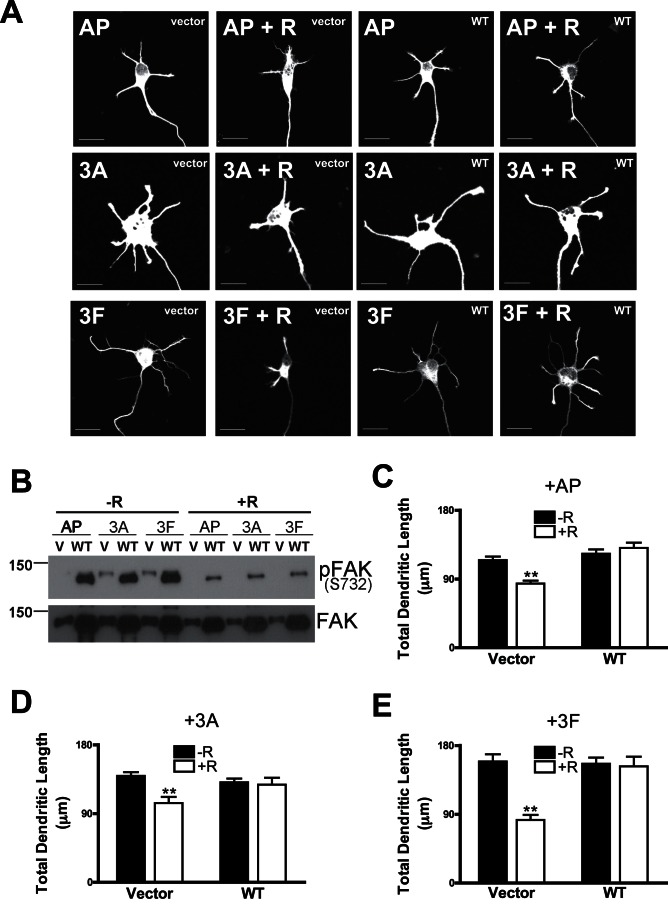
Roscovitine inhibition of Sema3A or 3F-induced dendritic growth was rescued by overexpression of wild-type FAK. (A) Representative images of primary hippocampal neurons transfected with vector or wild-type FAK (WT). Neurons were treated with AP control (AP), 3A-AP (3A), 3F-AP (3F), AP with roscovitine pre-treatment (AP+R), 3A-AP or 3F-AP with roscovitine pre-treatment (3A+R). Scale bar = 20 µm. (B) Representative blots showing phosphorylation of FAK in primary hippocampal neurons transfected with vector or WT-FAK treated with AP, Sema3A or Sema 3F together with vehicle (−R) or roscovitine (+R). (C, D and E) Graphs showing total dendritic length of primary hippocampal neurons expressing GFP (vector) or WT-FAK and treated with vehicle or roscovitine together with AP control (C), Sema3A (D) or 3F (E). 30–100 neurons from each group were used for analysis. Values represent mean ± s.e.m. (**P<0.01, 2-way ANOVA with Bonferroni’s post-hoc test).

**Figure 9:**
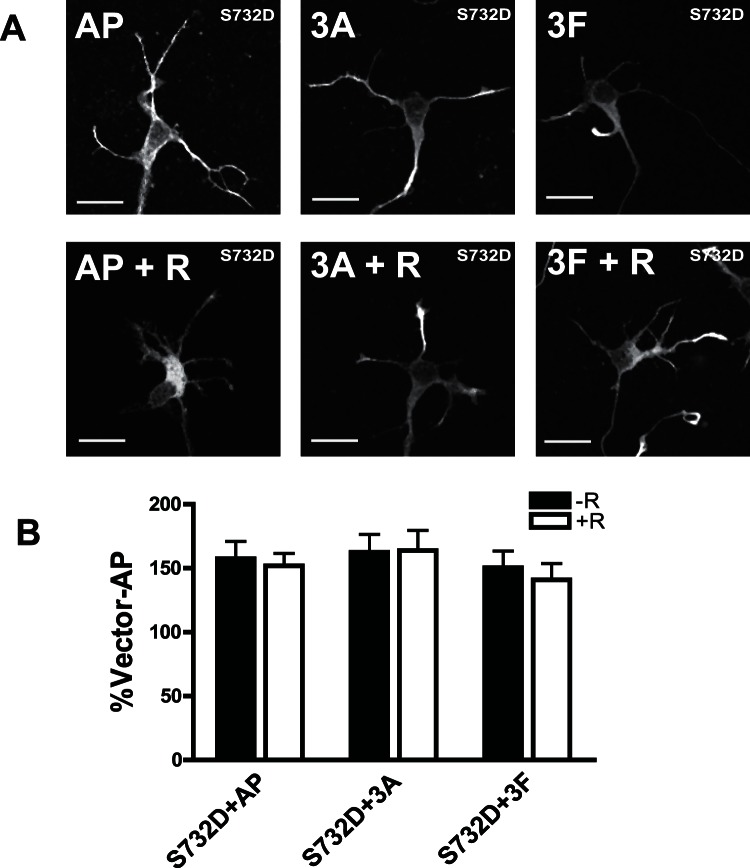
Overexpression of constitutively active FAK rescues the effects of roscovitine inhibition of semaphorin-induced dendritic growth. (A) Representative images of primary hippocampal neurons transfected with constitutively active FAK (S732D). Neurons were treated with AP control (AP), 3A-AP (3A), 3F-AP (3F), AP with roscovitine pre-treatment (AP+R), 3A-AP or 3F-AP with roscovitine pre-treatment (3A+R). Scale bar = 20 µm. (B) Graphs showing total dendritic length of primary hippocampal neurons expressing S732D FAK treated as indicated above. 40–50 neurons from each group were used for analysis. Values represent mean ± s.e.m. (No significant difference by 2-way ANOVA).

To address if Cdk5 mediates dendritic growth of Sema3A or 3F through FAK, we examined if activation of FAK could rescue the inhibition of dendritic growth by roscovitine in Sema3A or 3F treated neurons. We found that overexpression of wild type FAK could overcome the inhibition of dendritic growth by roscovitine in primary hippocampal neurons (Fig. 8A, C). Overexpressed WT FAK is highly phosphorylated at S732 in the basal state (without roscovitine) (Fig. 8B) but did not affect the dendritic length in basal state comparable to GFP (vector) expression in neurons (Fig. 8A,C). There was also residual S732 phosphorylation even after roscovitine treatment in the primary hippocampal neurons (Fig. 8A, B). This result indicated that WT FAK could possibly rescue the phenotype induced by Cdk inhibition. Treatment with Sema3A/3F increased dendrite length to a similar extent in GFP or wild-type FAK expressing neurons (Fig. 8D and E). Addition of roscovitine significantly reduced the total dendrite length of GFP expressing neurons (Fig. 8C, D and E). However, this reduction in dendritic length was rescued by overexpression of wild-type FAK (Fig. 8C, D and E). These results demonstrated that Cdk5-mediated dendritic growth stimulated by Sema3A or 3F through the activation of FAK. This rescue effect of WT FAK was further confirmed by another rescue experiment using constitutively active form of FAK (S732D FAK). Overexpression of S732D FAK overcame the effect of roscovitine on the inhibition of dendritic growth induced by Sema 3A and 3F (Fig. 9A and B). However, we noticed that compared with GFP vector, overexpression of S732D FAK itself increased the dendritic length in basal state but WT FAK does not affect the basal dendritic length of primary neurons (Fig. 8). To further prove that Cdk5 is upstream of FAK, we also performed rescue experiments using Cdk5 (Fig. S3). We expressed single serine mutant FAK-S732A in neurons and showed that dendritic growth induced by Semaphorins were abolished by this mutation in Figure 6. Overexpression of WT Cdk5 is not able to rescue the effect of this dominant negative FAK-S732A (Fig. S3). This result confirmed that Cdk5 is upstream of FAK in Semaphorin signaling pathway.

### FAK and Cdk5 are Downstream Mediators of Semaphorin Signaling in Newborn Neurons in the Adult CNS *in vivo*

**Figure 10:**
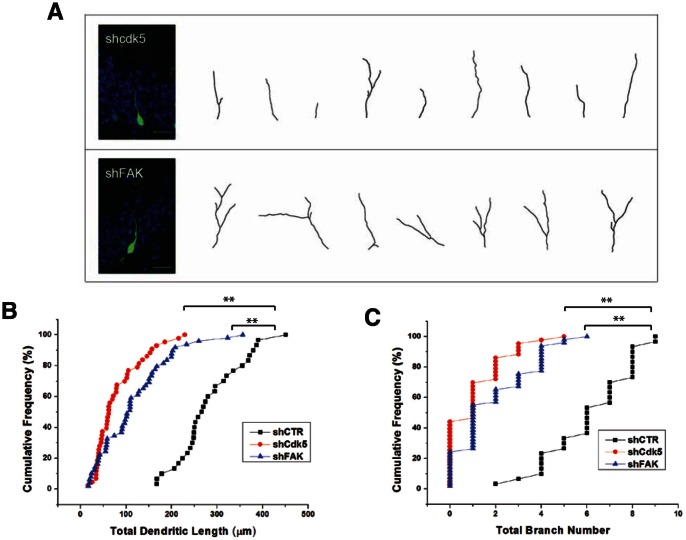
Knockdown of downstream mediators of semaphorin signaling, Cdk5 and FAK, induced defective dendritic morphologies of newborn DGCs in adult brain. (A) Representative images and tracings of dendrites of control, Cdk5 or FAK-shRNA expressing DGCs at 14 dpi (Scale bar, 20 µm). Graphs showing quantification of total dendritic length (B) and branch number (C) of newborn DGCs. Each symbol represents a single DGC at 14 dpi. (**P<0.01, 1-way ANOVA with Newman-Keuls’ post-hoc test).

**Figure 11:**
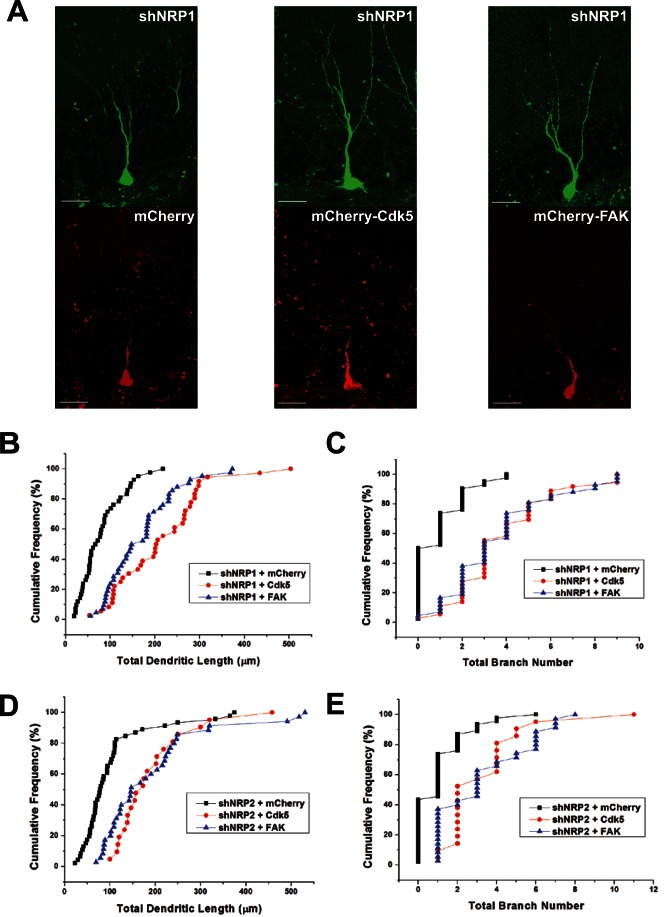
Overexpression of Cdk5 or FAK rescued dendritic phenotypes of NRP-1 and -2 deficient neurons. (A) Representative images of adult-born neurons co-infected with retrovirus expressing shNRP1 and mCherry shNRP1 and mCherry-T2A-Cdk5 and shNRP1 and mCherry-T2A-FAK. Scale bar = 20 µm. Quantification of total dendritic length (B, D) and branch number (C, E) of newborn DGCs at 14 dpi. Each symbol represents data from a single DGC at 14 dpi. (**P<0.01, 1-way ANOVA with Newman-Keuls’ post hoc test).

To determine if the effects of Cdk5 and FAK on dendritic development of cultured hippocampal neurons could also be observed in newborn neurons in adult CNS *in vivo*, we employed the same retroviral strategy for knocking down of Cdk5 and FAK in progenitor cells *in vivo*. Cdk5 or FAK deficiency resulted in impaired dendritic development of these newborn neurons (Fig. 10A, B and C), with a severity that is comparable to the knockdown of NRP1 or NRP2 (Fig. 2). This suggested that Cdk5 and FAK also played an important role in dendritic development of adult-born neurons *in vivo* and similar semaphorin signaling pathway might be conserved in embryonic neurons and newborn neurons in adult. To prove that semaphorin regulated dendritic development of adult-born neurons via activation of Cdk5 and FAK, we expressed Cdk5 or FAK in NRP1 or -2 deficient neurons (Fig. 11A–E). Overexpression of FAK or Cdk5 rescued the dendritic outgrowth phenotypes, by restoring (at least in part) the total dendritic length (Figure 11A, B and D) and branching (Figure 11A, C and E) of NRP1 or 2 silenced neurons. These evidences showed that Cdk5 and FAK were both downstream mediators of Sema3A/NRP1 and Sema3F/NRP2-induced dendritic development in newborn neurons in the adult CNS *in vivo*.

Taken together, these data demonstrated that semaphorins regulate the dendritic development of neurons in the novel context of the adult central nervous system. Importantly, we show that semaphorins induce both tyrosine and serine phosphorylation of FAK, and its consequent activation is critical for elaborating dendritic complexity in primary neurons *in vitro* and adult-born neurons *in vivo*.

## Discussion

Here we show Sema3A/NRP1 and Sema3F/NRP2 play an important role in dendritic development in newborn neurons in adult hippocampus. Knockdown of NRP1 and NRP2 affected both dendritic length and branch complexity of newborn neurons in adult hippocampus *in vivo*. It has been shown that defects in dendritic development of newborn granule neurons lead to defects in synapse formation and network integration of newborn neurons in adult, which are important in learning and memory [30], [31], [32], [33]. Defects in adult neurogenesis were also reported in many neurobehavioral disorders [34], [35]. Semaphorins have been implicated in spinal cord injury and various neurological disorders such as Rett syndrome, autism, epilepsy and Alzheimer’s disease, but the cellular basis and underlying mechanisms are still poorly understood [36], [37], [38], [39], [40], [41], [42]. Our study demonstrated an essential role of semaphorin signaling in adult neurogenesis and suggested the involvement of semaphorins in the etiology of neurobehavioral disorders.

Activation of FAK is required for chemorepellent or chemoattractant effects stimulated by multiple attractive and repulsive cues, such as semaphorins [43]. FAK functions as an important downstream mediator of semaphorin signaling during axonal remodeling and dendritic growth [15], [25], . Although semaphorin signaling induces tyrosine phosphorylation and subsequent activation of FAK, the involvement of FAK in dendritic growth is still controversial. Previous study and our results here show semaphorin signaling through FAK promotes an increase in total dendritic length and branch numbers [15], However, in contrast to these findings, increased in phosphorylated FAK (Tyr 397) mediated by protocadherin gamma (Pcdh**γ**) reduces dendritic growth in cortical pyramidal neurons *in vivo* [44]. Pharmacological inhibition of FAK through decreasing phosphorylation on Tyr 397, completely rescued this phenotype [44]. The knockout of the entire Pcdh**α** cluster also resulted in dendritic simplification through activating FAK (phosphorylation on Tyr 397) in CA1 pyramidal neurons *in vivo* and in cultured primary hippocampal neurons *in vitro* [45]. Notably, these two studies only examined FAK phosphorylation on Tyr 397. Therefore, the discrepancies on dendritic growth function of FAK could be attributed from the different cell types and also the different phosphorylation sites on FAK examined across different studies.

Tyrosine phosphorylation of FAK has been extensively studied, especially Tyr 397, which is the FAK autophosphorylation site and is the first event in FAK activation. FAK initiates its activation by autophosphorylating it’s Tyr 397 and triggering a series of tyrosine phosphorylation of FAK by other proteins including Src family kinases (SFKs) and integrin [46]. Functional outcome of FAK activation could be multifaceted, depending not only on the phosphorylation per se, but also largely dependent on the specific tyrosine residues that are being phosphorylated, the intensities and durations of the specific phosphorylation [26]. For example, Sema3A and netrin exert their opposing chemorepellent or chemoattractant effects possibly by differentially stimulating phosphorylation of FAK at different tyrosine sites. [26], [47], [48]. Our study here show Sema3A or 3F stimulates dendritic outgrowth through tyrosine phosphorylation of FAK at Tyr 397. Our data using tyrosine non-phosphorylated mutant of FAK confirmed this finding.

Interestingly, we found that a serine residue, Ser 732 of FAK also plays an important role in semaphorin-induced dendritic growth. We demonstrated that serine phosphorylation of FAK is mediated by activation of Cdk5. Serine phosphorylation of FAK is still not well studied, except that serine residue of FAK is involved in microtubule organization and neuronal migration in endothelial cells and neurons [49], [50]. Kinase activity of FAK is not directly regulated by serine phosphorylation, thus it is of interest to determine how serine phosphorylation affects cellular function of FAK. Our experiments using individual Tyr or Ser phosphorylation site mutants indicated that Tyr 397 and Ser 732 are regulated independently. Therefore, it is unlikely that serine phosphorylation regulates dendritic growth through tyrosine phosphorylation. However, we cannot completely exclude the possibility the functional interplay between tyrosine and serine phosphorylation of FAK because Ser 732 phosphorylation is known to affect Tyr 407 phosphorylation of FAK [51]. In addition, it is also possible that different extracellular cues or combination of different extracellular cues might lead to phosphorylation of FAK on different residues to promote different functions.

It has been reported that many functional events during neuronal development are recapitulated in adult neurogenesis. For instance, occurrence of GABA mediated-input before glutamatergic influence in developing neurons was observed in both embryonic and adult neurons [30]. It is fascinating that a classical embryonic guidance cue, such as semaphorins, can be co-opted to function in the adult context. Indeed, we found Cdk5 dependent FAK activation by semaphorin is conserved both in dendritic development of embryonic neurons and newborn neurons of adult, supporting conserved signaling pathways between embryonic development and adult neurogenesis. The various environmental and physiological cues encountered by the animal during adulthood are likely to affect the nature of neurogenesis. The precise regulation of semaphorin pathways during adulthood certainly deserves future lines of investigation.

## Materials and Methods

### Animals

Ethics statement: All animal procedures and applicable regulations of animal welfare were in accordance with IACUC guidelines and approved by Singhealth IACUC, Singapore.

Adult (5–6 weeks old) female C57Bl/6 mice and timed-mated Sprague-Dawley rats were purchased from Singhealth Experimental Medicine Centre (SEMC), Singapore, and housed in Specific Pathogen Free (SPF) animal facility at Duke-NUS Graduate Medical School, Singapore. All animals received water and food ad libitum. The surgical procedure was performed under anesthesia. 0.5% bupivacaine was administered locally after the surgery to provide temporary anesthesia to the wound. Additionally, after the stereotactic surgery, 1–5 mg/kg of butorphanol was administered subcutaneously for the first two days after surgery to help block pain from the surgical procedure. After this time, the animals should have little or no discomfort due to the wound healing. If animals continue to have pain and/or obvious discomfort outside this time period they were removed from the study and euthanized.

### Construction, Production and Stereotaxic Injection of Engineered Retroviruses

Engineered self-inactivating murine retroviruses were used to express GFP specifically in proliferating cells and their progeny as described previously [30]. GFP expression was under the control of EF1α promoter and shRNA was co-expressed under the control of human U6 promoter in the same vector. shRNAs against different regions of mouse NRP1-782: CGAATGTTCTCAGAACTAT, NRP1-2231: CACAGAGAAGCCAACCATT, NRP2-238: ACACGACTGCAAGTATGAC, NRP2-1076: TCGTACAAGCTGGAAGTCA, Cdk5-250: GATCAGGACCTGAAGAAAT, FAK-1412: GCCTTAACAATGCGTCAGT were cloned into retroviral vectors. Retroviral constructs encoding for cDNA of shRNA-resistant forms of NRP1 or NRP2, FAK or Cdk5-fused to mCherry via a T2A linker was driven by CAG promoter. Overexpression constructs encoding for cDNA of FAK (and FAK mutants), Cdk5 (and Cdk5 mutants) for primary hippocampal neurons were fused to mCherry via a T2A linker in FUGW vector.

To validate the specificity and efficiency of the shRNAs *in vitro*, retroviral vectors and expression constructs of myc-tagged mouse NRP-1 and -2 were co-transfected into HEK293T cells. NRP expression was determined by mouse anti-myc (Sigma, 1∶1000) with mouse anti-beta-tubulin (Sigma, 1∶10,000) as a loading control. High titers of engineered retroviruses (1×10^9^ unit/ml) were produced by co-transfection of retroviral vectors and VSVG into HEK293gp cells followed by ultracentrifugation. Adult mice were anaesthetized (100 µg ketamine, 10 µg xylazine in 10 µl saline per gram) and retroviruses were stereotaxically injected into the dentate gyrus as described previously [30]. Mice (4–6 animals per experiments) were sacrificed at 14 dpi for morphological analysis.

### Immunostaining, Confocal Imaging and Analysis

Coronal brain sections (40 µm) prepared from viral injected mice were processed for immunostaining. The following primary antibodies were used: goat anti-DCX (Santa Cruz, 1∶500), rabbit anti-Prox1 (Abcam, 1∶250). Images were acquired on a Zeiss LSM 710 confocal system (Carl Zeiss, Singapore) and analysed using Zeiss Zen software. For analysis of the dendritic structure of newborn neurons, three-dimensional (3-D) reconstructions of the dendritic processes of each GFP^+^ neuron were made from Z-series stacks of confocal images. The projection images were semi-automatically traced with NIH ImageJ using the NeuronJ plugin. Measurements do not include corrections for inclinations of dendritic process in 3-D and therefore represent projected lengths. The distributions of the total dendritic length and branch number of each individual neuron under different conditions were shown in accumulative distribution plots. A total of 25–30 neurons from 4–6 animals injected with shRNA were analyzed per group. Sholl analysis was used for analysing dendritic complexities of neurons. The number of dendrites crossing a series of concentric circles at 20 µm intervals from the cell soma was counted and presented as cumulative distribution plots. Statistical significance (*P*<0.05) was assessed using the student t-test.

### Primary Neuronal and Adult Neural Progenitor Cell (NPC) Cultures, Transfection and Immunofluorescence

Hippocampal neurons were isolated from the hippocampi embryonic rats (from timed-mated Sprague-Dawley as previously described [52]. Dissociated neurons were cultured on poly-l-lysine coated plates or coverslips. For biochemical analysis, neurons were treated with AraC to eliminate dividing astrocytes and used at 4–5 days after plating and starved in MEM without B27 supplements 2–3 hrs prior treatment with Sema3A/3F. Primary neurons were transfected with the Amaxa electroporation device using a nucleofection method before plating. After immunostaining, images were acquired on a Zeiss LSM 710 confocal system (Carl Zeiss, Singapore) and images were semi-automatically traced with NIH ImageJ using the NeuronJ plugin. As MAP2 and Tau expression were not strong in neurons at 3 DIV, axons (longest thin process) and dendrites (shorter, tapering processes) were assessed by their well-defined morphologies after staining with anti-DCX antibody. More than 50 neurons were traced in each experiment and were repeated at least three times. Adult neuroprogenitor cells were isolated from 6 week old C57Bl/6 mice as previously described [53].

### Production of Alkaline Phosphatase (AP) Fused Semaphorin (Sema-AP) Ligands

To produce AP-fused Sema3A and 3F ligands, HEK cells were transfected with constructs encoding Sema3A-AP and Sema3F-AP. After 24 hr post transfection, media was replaced with Serum free HEK 239 Freestyle media (Invitrogen) and cells were growth for a further 48 hrs. The media was concentrated and the amount of secreted ligands is quantified with a standard diethanolamine assay.

### Cell Lysis and Immunoblotting

HEK293T cells were cultured in Dulbecco’s modified Eagle’s medium supplemented with 10% fetal bovine serum (FBS) and were transfected with the indicated plasmids using calcium phosphate method. For biochemical experiments, HEK293T cells were starved for 16 hrs prior to treatment. Primary neurons and HEK293T cells were stimulated with AP, Sema3A and Sema3F for 15 min. Primary neurons were pretreated with roscovitine 1 hr prior to 15 min of ligand stimulation. Total lysates from these cells and primary neurons were prepared using RIPA buffer (1% Triton X-100, 50 mM HEPES, pH 7.0, 150 mM NaCl, 2 mM EGTA, 0.25% sodium deoxycholate, 10 mM NaF, 1 mM Na_3_VO_4_, 0.2 mM phenylmethylsulfonyl fluoride, 10 µg/ml pepstatin, 10 µg/ml aprotinin, 10 µg/ml leupeptin). The following primary antibodies were used: rabbit anti-phospho-FAK (Tyr397) (Cell Signaling Technology, 1∶1000), rabbit anti-phospho-FAK (Ser732) (Abcam, 1∶1000), mouse anti-FAK (Millipore, 1∶5000), mouse anti-myc (Sigma, 1∶1000), mouse anti-Tubulin (Sigma, 1∶10000), rabbit anti-phospho-DCX (Cell Signaling Technology, 1∶1000), rabbit anti-phospho Paxillin (Cell Signaling Technology, 1∶1000), mouse anti-Paxillin (Millipore, 1∶1000).

### Statistical Analysis

GraphPad Prism Software was used for all statistical analyses. Statistical significance between three or more groups was analyzed using a one-way analysis of variance (ANOVA) with Newman-Keuls’ post-hoc tests. For interaction analysis, a two-way AOVA was used and the interaction term was determined.

## Supporting Information

Figure S1 Validation of the efficiency of NRP1/NRP2 shRNA knockdown in primary hippocampal neurons. E18 hippocampus neurons were electroporated with shCTR (A, C), shNRP1 (B) and shNRP2 (D) constructs. Neurons were fixed at 6 DIV and immunostained with anti-NRP1 and anti-NRP2. Representative images show effective knockdown of endogenous NRP1 (B) and NRP2 (D) in primary neurons. White arrows indicate GFP-shRNA positive neuronal cell bodies stained with anti-NRP1 or NRP2 antibodies as indicated. White arrowheads indicate dendrites.
(EPS)

Figure S2 Characterization of retrovirus labeled newborn cells in the adult DG. Retrovirus labeled GFP-positive cells express DG granular neuronal marker, Prox1 (red) and immature neuronal marker, DCX (pink). White arrows indicate colocalizations of different markers. Scale bar = 10 µm.
(EPS)

Figure S3 Serine phosphorylation of FAK by Cdk5 is important for Sema3A or 3F-induced dendritic growth. (A) Graphs showing total dendritic length of primary hippocampal neurons expressing single serine dominant negative mutant FAK-S732S together with control vector (mcherry) or with Cdk5-WT treated with AP control (top), Sema3A (middle) or 3F (bottom). 30 neurons from each group were used for analysis. Values represent mean ± s.e.m.
(EPS)
